# Maternal Obesity and Differences in Child Urine Metabolome

**DOI:** 10.3390/metabo14110574

**Published:** 2024-10-25

**Authors:** Ellen C. Francis, Kelly J. Hunt, William A. Grobman, Daniel W. Skupski, Ashika Mani, Stefanie N. Hinkle

**Affiliations:** 1 Department of Biostatistics & Epidemiology, Rutgers School of Public Health, Piscataway, NJ 08854, USA; ellen.francis@rutgers.edu; 2 Public Health Sciences, Medical University of South Carolina, Charleston, SC 29425, USA; huntke@musc.edu; 3 Department of Obstetrics and Gynecology, Brown University Medical School, Providence, RI 02903, USA; grobman.7@osu.edu; 4 Weill Cornell Medicine, New York Presbyterian Queens, Flushing, NY 11355, USA; dwskupsk@med.cornell.edu; 5 Department of Biostatistics, Epidemiology and Informatics, Perelman School of Medicine, University of Pennsylvania, Philadelphia, PA 19104, USA; ashika.mani@pennmedicine.upenn.edu; 6 Department of Obstetrics and Gynecology, Perelman School of Medicine, University of Pennsylvania, Philadelphia, PA 19104, USA

**Keywords:** pregnancy, developmental origins, metabolomics, microbiome, obesity, child, urine

## Abstract

**Background/objective:** Approximately one-third of pregnant individuals in the U.S. are affected by obesity, which can adversely impact the in utero environment and offspring. This study aimed to investigate the differences in urine metabolomics between children exposed and unexposed to maternal obesity. **Methods:** In a study nested within a larger pregnancy cohort of women–offspring pairs, we measured untargeted metabolomics using liquid chromatography–mass spectrometry in urine samples from 68 children at 4–8 years of age. We compared metabolite levels between offspring exposed to maternal obesity (body mass index [BMI] ≥ 30.0 kg/m^2^) vs. unexposed (maternal BMI 18.5–24.9 kg/m^2^) and matched them on covariates, using two-sample t-tests, with additional sensitivity analyses based on children’s BMI. This study reports statistically significant results (*p* ≤ 0.05) and potentially noteworthy findings (fold change > 1 or 0.05 < *p* < 0.15), considering compounds’ involvement in common pathways or similar biochemical families. **Results:** The mean (SD) maternal age at study enrollment was 28.0 (6.3) years, the mean child age was 6.6 (0.8) years, 56% of children were male, and 38% of children had a BMI in the overweight/obese range (BMI ≥ 85th percentile). Children exposed to maternal obesity had lower levels of 5-hydroxyindole sulfate and 7-hydroxyindole sulfate and higher levels of secondary bile acids. Phenylacetic acid derivatives were lower in offspring exposed to obesity and in offspring who had a current BMI in the overweight/obese range. Exposure to maternal obesity was associated with lower levels of androgenic steroid dehydroepiandrosterone sulfate (DHEA-S). **Conclusions:** In this preliminary study, children exposed to maternal obesity in utero had differences in microbiome-related metabolites in urine suggestive of altered microbial catabolism of tryptophan and acetylated peptides. Some of these differences were partially attributable to the offspring’s current BMI status. This study highlights the potential of urine metabolomics to identify biomarkers and pathways impacted by in utero exposure to maternal obesity.

## 1. Introduction

Approximately one-third of pregnant people in the U.S. are affected by obesity [1]. Living with obesity puts individuals at an increased risk of developing gestational diabetes and hypertensive disorders of pregnancy [2]. Excessive maternal weight, both in the presence and absence of comorbidities [3], can adversely affect the in utero environment. Specifically, excess adiposity, mainly visceral, can lead to increased levels of inflammation, oxidative stress, and insulin resistance [4,5]. Additionally, pregnant people with obesity often experience high levels of physiological stress from weight stigma and bias, which also has the potential to alter the intrauterine environment [3]. Consequently, there is a higher risk of negative short- and long-term health outcomes for offspring, including preterm birth and large-for-gestational-age birthweight, as well as childhood obesity and metabolic diseases [6]. Many human and animal studies support the lasting impact of the in utero environment on health throughout the life course [6,7].

Advances in high-throughput technologies have improved our understanding of the biological pathways by which the maternal phenotype can impact offspring health. These studies have highlighted changes in placental function [8,9,10,11], epigenetic modification of metabolism-related genes measured in umbilical cord blood [12,13,14], and variations in metabolites of steroid hormones, branched-chain amino acids, and carnitines in children’s blood [15,16,17,18]. However, alterations in pathways related to exposure to maternal obesity in utero remain less explored in easily obtainable biological samples such as urine.

Metabolomics is becoming commonly used to help identify metabolic pathways and markers that are altered by exposures due to the ability of metabolomics to reflect environmental, genetic, and proteomic interactions, and as such can be a sensitive measure of the organism’s phenotype [19]. Notably, metabolomics offers the potential to uncover changes in maternal metabolism during pregnancy and their relationship to the metabolic state of the offspring from the newborn state [20]. Urine is an easily obtainable specimen, and as such may be useful to understand metabolic changes in children after an in utero exposure, such as maternal obesity. Metabolites detected in urine reflect endogenous processes such as lipid metabolism and insulin resistance, as well as exogenous influences such as dietary intake and gut microbial activity [21,22,23,24]. For instance, studies have shown that obesity is associated with differences in acylcarnitines in urine, which likely reflect upstream impairments in fatty acid beta-oxidation, a key process in energy homeostasis [25]. Yet, a 2021 review of metabolomic studies of childhood obesity identified 33 studies measuring metabolomics in blood, while only 4 utilized urine [18]. In the studies that used blood, there was a general consensus that children with obesity exhibited elevated levels of amino acids (especially branched-chain and aromatic), carnitines, lipids, and steroids. In the studies that used urine, the metabolomics findings generally aligned with those from blood, although additional differences were noted in carbohydrate, diet, and gut microbial activity [18,26,27,28]. Of the studies that measured metabolomics in urine, two were among adolescent participants (one conducted in the US [29] and one in Korea [30]), and two were among children participants from European countries [26,27]. Given the differences in diet and environmental exposures between the US and European countries, and the rapid growth and development that occurs in puberty, there remains a gap in the literature with respect to the urine metabolome of children. Further, there are ethical and practical challenges in obtaining blood samples from children, and therefore, exploring urine as a metabolomic matrix is crucial. Utilization of urine for metabolomic studies in children will allow us to better understand the effects of exposure to obesity in utero as well as other important environmental exposures such as maternal diet, physical activity, and chemicals.

The aim of this US-based preliminary study was to compare metabolomics measured from the urine of children unexposed versus exposed to maternal obesity, which could inform future targeted studies focused on identifying markers and differences in biochemical pathways in children.

## 2. Methods

### 2.1. Participants and Settings

The *Eunice Kennedy Shriver* National Institute of Child Health and Human Development (NICHD) Fetal Growth Studies—Singletons was a prospective cohort study of pregnant women conducted at 12 U.S. clinical centers. Briefly, recruitment was conducted between 2009 and 2013 and included participants aged 18–40 years enrolled at 8 to 13 weeks of gestation, who were then followed through delivery. Eligibility was limited to those with a pre-pregnancy BMI of 19.0–45.0 kg/m^2^ who generally were without pre-existing chronic conditions or behavioral factors that may impact fetal growth [31]. At enrollment, women were interviewed based on standardized and structured questionnaires to collect information on their demographic characteristics and reproductive and pregnancy history. Maternal pre-pregnancy weight, self-reported at enrollment, and height, measured at enrollment, were used to calculate pre-pregnancy BMI, which was categorized as normal weight (BMI 18.5–24.9 kg/m^2^), overweight (BMI 25.0–29.9 kg/m^2^), and obesity (BMI ≥ 30.0 kg/m^2^). All participants provided written informed consent.

The Environmental Influences on Child Health Outcomes (ECHO) Fetal Growth Study was a follow-up of the children of the maternal participants from 10 of the clinical centers within the NICHD Fetal Growth Studies—Singletons [32]. Children were invited for follow-up between May 2017 and April 2019; as such, children ranged in age from 4 years to 8 years at the follow-up. Children participated in a clinical exam during which urine was collected, structured questionnaires were completed, and anthropometry was measured. Children’s weight and height were measured at least twice during the clinical assessment [32]. Age- and sex-adjusted BMI (kg/m^2^) percentiles were calculated using the 2000 US CDC Growth Charts. BMI (kg/m^2^) percentile was categorized as normal weight (<85th percentile), overweight (85th to <95th percentile), or obese (≥95th percentile).

For the current study, stored urine samples were randomly selected for a sample of mothers with obesity and a matched sample of mothers with normal weight. Mothers categorized as overweight were not included in this preliminary study of urinary metabolomics. Each randomly selected individual with obesity was then matched to one with normal weight based on an exact match for child sex and a propensity score to control for potential confounding related to obesity. The variables used to generate the propensity score included maternal age (18–24, 25–29, 30–34, ≥35 years), self-reported race/ethnicity (Asian/Pacific Islander, Hispanic, Non-Hispanic Black, Non-Hispanic White), parity (0, ≥1), education (high school or less, some college or associate degree, four-year college degree or higher), and child sex (female, male) and age (continuous). Only samples of non-smoking women were included as smoking was an exclusion criterion for women with a BMI of 19–29.9 kg/m^2^ in the main study. A sample size of 70 (35 exposed/35 unexposed) was chosen based on the budget for this preliminary work. The analysis included 68 mother–child pairs (Supplemental Figure S1); there was an error in the sample selection for one of the matched pairs and metabolomics were not assessed on this pair and consequently excluded from the analysis.

Approval for the baseline data collection was received from the Institutional Review Board (IRB) at each participating site, the data coordinating center, and the NICHD. All mothers provided written informed consent. The ECHO follow-up was approved by the Medical University of South Carolina IRB and a Central IRB at Columbia University Medical Center and by all the participating sites. Written informed consent was obtained from the parent or legal guardian of each enrolled child. Depending on the child’s age and state regulations, child assent was obtained. Approvals for this analysis of the children’s stored urine samples were obtained by the Central IRBs at the Columbia University Medical Center and the University of Pennsylvania.

### 2.2. Metabolomics

Children’s urine was stored as 2.0 mL aliquots in external thread polypropylene urine storage vials with a sealing gasket. Untargeted metabolome analysis of childhood urine was performed by Metabolon© (Morrisville, NC, USA). Details on the acquisition, data preprocessing, and annotation of metabolomics data are provided in the Supplementary Materials. Briefly, data were acquired on a Waters ACQUITY ultra-performance liquid chromatography (UPLC) and a Thermo Scientific Q-Exactive high-resolution (Waltham, MA, USA)/accurate mass spectrometer interfaced with a heated electrospray ionization (HESI-II) source and an Orbitrap mass analyzer operated at 35,000 mass resolution [33]. The UPLC-MS/MS data were processed using a combination of Metabolon-developed software services (applications). Peaks detected by UPLC-MS/MS were identified or annotated by matching them to an in-house physical standard library.

### 2.3. Statistical Analysis

In the primary analysis, we used a two-sample t-test to assess mean differences in urine metabolites between offspring unexposed versus exposed to maternal obesity. Due to the preliminary nature of this study, and to provide rich data for future studies to explore further, we report results for potentially noteworthy findings which are tests that achieved statistical significance (*p*-value ≤ 0.05), and tests where the fold change was >1 or the *p*-value was approaching significance (0.05 < *p*-value < 0.15). In reporting differences, and whether a compound might warrant further study, we take into account whether the compound is included in a common pathway with a highly significant compound, or if it is in a similar functional biochemical family with other significant compounds.

In sensitivity analyses, we compared any differences observed in the primary analysis with findings from a two-sample t-test of mean differences between groups based on the child’s BMI (normal range vs. overweight/obese range). Additionally, we used weighted correlation network analysis (WGCNA) to capture the complex interplay between compounds. Details on the WGCNA algorithm and nomenclature have been described elsewhere [34]. Briefly, the algorithm constructs a network by first generating an adjacency matrix, which uses Pearson correlations (R2) to measure the connectedness between compounds. The adjacency matrix is then transformed to a topological overlap matrix which measures the connectedness between compounds considering their relationship to all other compounds within the network and subsequently performs hierarchical clustering on the topological overlap matrix based on dissimilarity. We used a network preservation approach, which creates a network in a particular group such as a healthy or reference group, and then superimposes the network onto a comparison group, such as offspring exposed to maternal obesity. We created the network in the reference group which was either unexposed to maternal obesity or offspring with a BMI in the normal range at follow-up. We compared the preservation of the reference network in both exposure settings (maternal pre-pregnancy BMI or child BMI status) to exposed offspring (i.e., offspring of women with obesity or offspring who themselves had a BMI in the overweight/obese range, respectively) using a quantitative measure of network preservation developed for the WGCNA package. The quantitative measure of network preservation aggregates several module preservation statistics into a composite preservation z-summary score, with scores > 10 indicating high preservation [35]. Analyses were completed using SAS (v. 9.4, Cary, NC, USA) and R (v4.1.2; R Core Team 2021).

## 3. Results

The full characteristics of the sample are shown in Table 1, which demonstrates the balanced covariates by design according to maternal normal weight (n = 34) and obesity (n = 34) status. The mean (SD) maternal age at study enrollment was 28.0 (6.32), the mean child age was 6.6 (0.8), 56% of children were male, and 38% of children had a BMI in the overweight/obese range (BMI ≥ 85th percentile). The metabolomics data included 1510 compounds, with 1028 compounds of known identity (named compounds), and after excluding xenobiotics, cofactors, vitamins, and those with >50% missing across samples, 662 compounds were included in our analysis.

### 3.1. Primary Analysis

**Figure 1:**
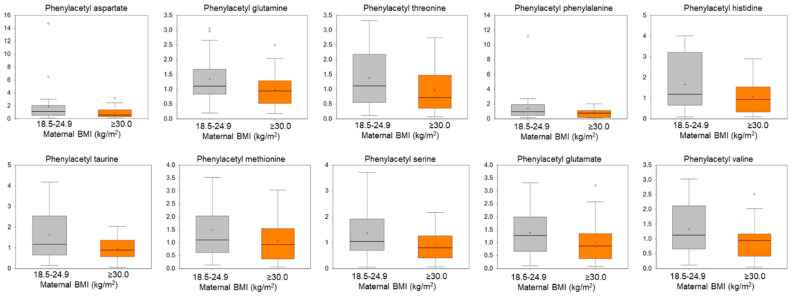
Mean differences in acetylated peptides measured in offspring urine during childhood. Orange boxplots show the distribution of acetylated peptides in urine among offspring exposed to maternal obesity in utero. Gray boxplots show the distribution of acetylated peptides in urine among offspring unexposed to maternal obesity. Pre-pregnancy maternal BMI is categorized by normal weight (BMI 18.5–24.9 kg/m^2^) and obesity (BMI ≥ 30.0 kg/m^2^).

Exposure to maternal obesity was associated with urine metabolome differences in microbiome-related metabolites, acetylated peptides, steroid metabolites, and some carbohydrate metabolites (Supplemental Table S1). With respect to microbiome-related metabolites, we found that 5-hydroxylindole sulfate (fold change 0.61, *p*-value = 0.13) and 7-hydroxyindole sulfate (fold change 0.43, *p*-value = 0.02) were lower in the exposed compared with the unexposed offspring. Phenylacetyl metabolites were all lower in offspring exposed to obesity vs. unexposed (range of fold changes: 0.50–0.73; range of *p*-values: 0.03–0.10) (Figure 1). We also found that the primary bile acid cholate was substantially higher (fold change 4.77, *p*-value = 0.03) in the exposed offspring compared to unexposed, as were the secondary bile acids glycolithocholate sulfate (fold change 0.58, *p*-value = 0.01) and 7-ketodeoxycholate (fold change 2.80, *p*-value = 0.11).

Androgenic steroid metabolites were lower in the exposed offspring compared to offspring unexposed, such as 11beta-hydroxyandrosterone sulfate (fold change 0.42, *p*-value = 0.06) which is produced from cholesterol. Other steroid hormones, cortisol, cortisone, and the detected corticosteroid metabolites were not different between exposed offspring compared with the unexposed. The lactose-related amino sugar 3′-a-sialyl-N-acetyllactosamine was higher among exposed offspring (fold change 1.44, *p*-value = 0.09).

**Figure 2:**
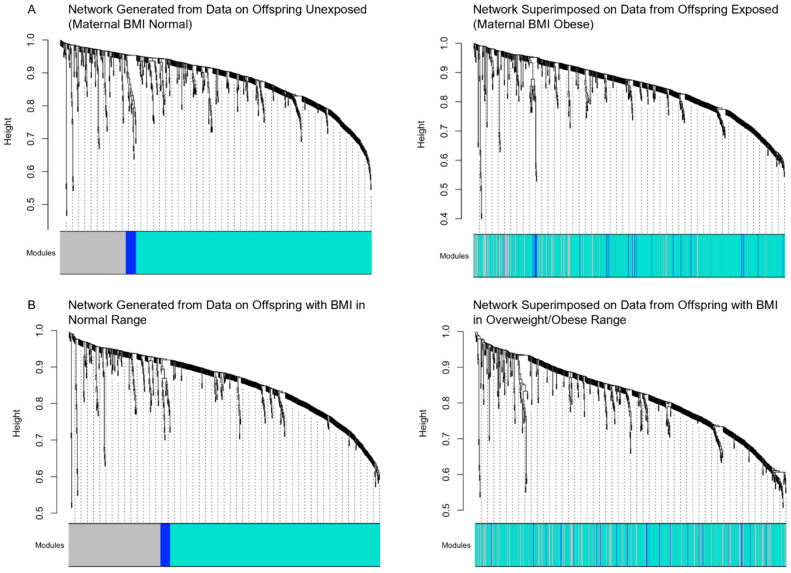
**Preservation of metabolite network modules in offspring urine.** Module preservation is used to assess the biological relevance of correlated metabolites in modules. Poor preservation can occur when the interrelatedness of metabolites differs between “reference/control” and “case/disease” samples. In our study, the reference group was based on BMI status—either exposure to maternal normal BMI range (**A**), or offspring normal BMI range in childhood (**B**). Modules with good preservation indicate that the interrelatedness of metabolites is similar in reference and case samples. Poor module preservation indicates that the interrelatedness of metabolites is not preserved in the case samples. Preservation is assessed qualitatively by superimposing the network derived in the reference group onto the cases, as well as quantitatively using a series of different preservation metrics that are summarized in a z-summary score. Metabolites assigned to the Gray module are considered unassigned. (**A**) The panel on the left shows the reference network and the two modules identified (Blue and Turquoise). The right panel shows the network after it is superimposed onto the urine metabolomics data from offspring exposed to obesity. Overall, the Turquoise cluster was well preserved, while the Blue cluster was poorly preserved. (**B**) This row of figures shows the metabolomic network derived using data from urine metabolomics in offspring who had a BMI in the normal range at the time of urine sampling (on the left panel). The right panel shows the network after it is superimposed onto the urine metabolomics data from offspring who had a BMI in the overweight/obese range at the time of urine sampling. Overall, the Turquoise cluster was well preserved, while the Blue cluster was poorly preserved.

The weighted correlation network analysis that was derived among offspring of mothers with normal weight resulted in two clusters of compounds (Figure 2: panel A): (1) a large Turquoise cluster that included 400 of the named compounds from metabolic pathways of amino acid (lysine, tryptophan, arginine, proline, and taurine metabolism), polyamine, and creatine metabolism, and nucleotide (RNA) turnover (Supplemental Table S2); and (2) a small Blue cluster of 21 short-chain carnitines reflective predominantly of fatty acid metabolism. This smaller Blue cluster was poorly preserved in the offspring exposed to maternal obesity (z-summary score = 3.2).

### 3.2. Sensitivity Analyses Based on Children’s BMI Exposure

When we compared the differences in metabolite levels based on offspring BMI status, we found that for the indole 7-hydroxyindole sulfate, and the phenylacetyl metabolites phenylacetylglutamine and phenylacetylphenylalanine, the differences observed based on exposure to maternal obesity were also apparent based on the child’s current BMI categorization. When we derived the reference metabolomic network in the group of offspring with a BMI in the normal range, the network cluster composition and metabolites were almost identical to the cluster composition identified when the reference network was generated using data from the offspring unexposed to maternal obesity (Figure 2: panel B).

## 4. Discussion

The aim of this preliminary study (n = 68) was to compare urine metabolomics measured from children at approximately 6 years of age, half of whom were exposed to maternal obesity in utero. Our findings highlight differences in urine metabolomics in microbiome-related metabolites suggestive of altered microbial catabolism of tryptophan and phenylalanine, and acetylated peptides such as phenylacetyl amino acids. These data indicate possible altered gut microbial activity in childhood among offspring exposed to maternal obesity and could be further explored in future targeted studies investigating markers and/or the impacts of the in utero environment across early life.

In the proceeding sections, we discuss observed differences based on our criteria of *p*-values and fold change and provide context for the related metabolic pathways. We note that the present results were from an exploratory study and should be interpreted cautiously. Our discussion below of the relationship to biological processes may capture only some of the possible explanations for these differences. Moreover, there is a strong correlation between maternal and child obesity, and thus, some of the differences may be an artifact of the child’s own elevated BMI. To address this, any differences observed following exposure to maternal obesity were further analyzed by offspring BMI exposure groups. We interpret consistent differences between maternal and offspring BMI exposure groups as associations that are more likely attributable to offspring BMI, whereas in terms of differences only present following exposure to maternal obesity, we interpret them as more likely to be attributable to in utero exposure.

Predominately, our findings suggest differences in microbiome-related metabolites based on differences in amino acid and short-chain fatty acid metabolites. We found that 5-hydroxylindole sulfate and 7-hydroxyindole sulfate were both lower in the offspring exposed to maternal obesity compared with the unexposed offspring. Indole arises from microbial fermentation of tryptophan, which can then be modified by sulfation and hydroxylation [36]. The lower levels of microbial amino acid metabolites in the exposed offspring do not appear to be the result of lower tryptophan, as it was not different between the two groups. We also found that the primary bile acid cholate was substantially higher in the offspring exposed to maternal obesity compared to unexposed. Several other primary bile acid metabolites were elevated, and this could suggest increased bile acid production in the exposed offspring. Differences in cholate and other secondary bile acids such as glycolithocholate sulfate and 7-ketodeoxycholate levels without concomitant differences in glycocholate may indicate altered microbial modification of bile acids [37]. Given that the gut microbiome plays a vital role in primary and secondary bile acid metabolism and catabolizes dietary components like tryptophan, these data may possibly point to an altered composition of the microbiome. Although these observations may partially reflect the offspring’s own BMI status, we did not find similar differences in our sensitivity analysis based on the offspring’s current BMI. Apart from 7-hydroxyindole sulfate, the differences in these short-chain fatty acids and amino acids were reflective of exposure to maternal obesity, and the data did not indicate that the differences were a product of the offspring’s current BMI.

The majority of measured phenylacetyl amino acids captured in the metabolomic assay were lower in offspring exposed to maternal obesity. Only phenylacetylisoleucine did not achieve our criteria for indicating a difference (fold change 0.70, *p*-value = 0.15). Our sensitivity analysis indicates that some of these differences may be attributed to the offspring’s current BMI status, as phenylacetylglutamine and phenylacetylphenylalanine were also lower in offspring with a BMI in the overweight/obese range compared to offspring with a BMI in the normal range. Interestingly, although we found lower levels of phenylacetylglutamine, there were no concomitant changes observed with urea, suggesting that it is unlikely that alterations to the urea cycle underlie the changes in phenylacetylglutamine. The lower levels of phenylacetyl amino acids could possibly result from decreased conversion of phenylalanine to phenylacetate by the gut microbiota [38]. Alternatively, it may be that liver phenylacetyl transferase or N-acetyl transferase activity is different among offspring exposed to obesity, which could impact the formation of phenylacetyl amino acids [39,40].

Some androgenic steroid metabolites were lower in the offspring exposed to maternal obesity. In particular, we observed that dehydroepiandrosterone sulfate (DHEA-S) was lower in the exposed compared with unexposed. DHEA is produced from cholesterol and functions as a precursor to both testosterone and estrogen, as well as pregnenolone and cortical steroids [41]. Interestingly, metabolites of pregnenolone steroids were not altered. It may be that DHEA is being utilized to a greater extent for cortisol production. This aligns somewhat with prior findings on elevated cortisol levels among individuals with obesity [42]; however, we did not find significant differences in sensitivity analysis when groups were compared based on the offspring’s own BMI in childhood, which may indicate some lasting influence of in utero exposure to maternal obesity. Finally, we observed higher levels of the lactose-related amino sugar 3′-a-sialyl-N-acetyllactosamine, which could be suggestive of some degree of greater intestinal permeability in obesity-exposed offspring [43].

Apart from some mothers having a high BMI, the sample of mother–offspring pairs in this preliminary study was otherwise healthy. In our sensitivity analysis using weighted correlation networks, we found that regardless of whether the reference network was derived in offspring not exposed to obesity, or among offspring who themselves had a normal range BMI, the interrelationship between compounds in the network was largely similar between groups. These similarities in the metabolomic network, regardless of how the groups were categorized (maternal normal BMI vs. child normal BMI), reflect the overall stability in the metabolome among an otherwise healthy pregnant and child population. Additional explanations might be related to a somewhat minimal impact of maternal obesity without other pre-existing comorbidities on the urine metabolome of offspring in early childhood. Considering that metabolic processes primarily occur within cells, future studies examining the impact of in utero exposure to maternal obesity on offspring health might gain greater insight by assessing metabolic activity across various tissue matrices, although these samples are much more difficult to obtain in children. Nevertheless, this approach could help triangulate data to identify disrupted pathways or efficient biomarkers.

## 5. Limitations

In this study, we reported on differences based on less stringent criteria for *p*-values and focused on the pathways with differences in multiple metabolites and in similar directions. However, due to the small sample size of our study, we cannot rule out the possibility that some differences are due to random chance. Conversely, we do not want to ignore potential interesting findings for future studies to explore simply because mean differences did not meet the usual thresholds of statistical significance (*p*-value < 0.05). We caution the reader to interpret these data with care and encourage future studies to explore temporal changes, sex-based differences, and targeted metabolomics platforms in multiple tissues concurrently.

## 6. Conclusions

This preliminary study compared the urine metabolome of offspring from women with pre-pregnancy obesity versus the metabolome of those with normal BMI status. Our findings highlight some differences in microbiome-related metabolites, acetylated peptides, and steroid metabolites, with the most notable differences indicative of gut-microbiome function. Future studies could leverage these comprehensive data for the further targeted study of the composition and diversity of the gut microbiome of children based on in utero exposure, and to understand some of the potential pathways by which maternal exposure impacts the health of offspring across their early life.

## Figures and Tables

**Table 1:** Sample characteristics overall and by pre-pregnancy weight status.

		Pre-Pregnancy Weight Status		*p*-Value
	Overall	Normal Weight	Obesity	
	n = 68	n = 34	n = 34	
**Age of Mother**				0.26
Mean (SD)	28.0 (6.3)	27.1 (6.5)	28.9 (6.1)	
**Maternal Education Status**				0.83
≤High School	20 (29.4%)	9 (26.5%)	11 (32.4%)	
Some College/Associate Degree	24 (35.3%)	12 (35.3%)	12 (35.3%)	
≥4-Year College Degree	24 (35.3%)	13 (38.2%)	11 (32.4%)	
**Maternal Race**				0.70
Non-Hispanic White	18 (26.5%)	9 (26.5%)	9 (26.5%)	
Non-Hispanic Black	27 (39.7%)	15 (44.1%)	12 (35.3%)	
Hispanic	23 (33.8%)	10 (29.4%)	13 (38.2%)	
Asian/Pacific Islander	0 (0%)	0 (0%)	0 (0%)	
**Parity**				0.96
0	27 (39.7%)	13 (38.2%)	14 (41.2%)	
1	29 (42.6%)	15 (44.1%)	14 (41.2%)	
2	12 (17.6%)	6 (17.6%)	6 (17.6%)	
**Sex of Child**				1.00
Male	38 (55.9%)	19 (55.9%)	19 (55.9%)	
Female	30 (44.1%)	15 (44.1%)	15 (44.1%)	
**Age of Child**				0.17
Mean (SD)	6.6 (0.8)	6.7 (0.8)	6.5 (0.8)	
**Child BMI Percentile**				0.002
Mean (SD)	68.3 (29.4)	57.5 (29.3)	79.1 (25.5)	
**Child Weight Status**				0.01
Normal Weight	42 (61.8%)	26 (76.5%)	16 (47.1%)	
Obesity	26 (38.2%)	8 (23.5%)	18 (52.9%)	

## Data Availability

The authors of the paper do not have the authority to share the data per their Data Use Agreements. Those interested in the data should contact the NICHD. https://www.nichd.nih.gov/about/org/dir/dph/officebranch/eb/fetal-growth-study (accessed on 10 August 2024).

## Supplementary Materials

The following supporting information can be downloaded at https://www.mdpi.com/article/10.3390/metabo14110574/s1: Supplemental Figure S1: Sample selection; Supplemental Table S1: Observed Biochemical Differences in Offspring Urine Metabolome by Maternal Obesity Status; Supplemental Table S2: Weighted correlation network analysis results.
