# Supplementary material for: Maternal Obesity and Differences in Child Urine Metabolome
Source: Metabolites. 2024 Oct 25;14(11):574. doi: 10.3390/metabo14110574 (PMC11596954; doi:10.3390/metabo14110574)
Supplement: Supplementary file 1 [file metabolites-14-00574-s001.zip › Francis Supplemental Material 2024_08_24.pdf]

Supplemental Material

Maternal Obesity and Differences in Child Urine Metabolome

Ellen C. Francis, Kelly J. Hunt, William A. Grobman,

Daniel W. Skupski, Ashika Mani, Stefanie N. Hinkle

Supplemental Figure 1	Page 2
Supplemental Table 1	Excel Sheet
Supplemental Table 2	Page 3
Supplemental Methods	Page 5

Supplemental Figure 1. Sample selection

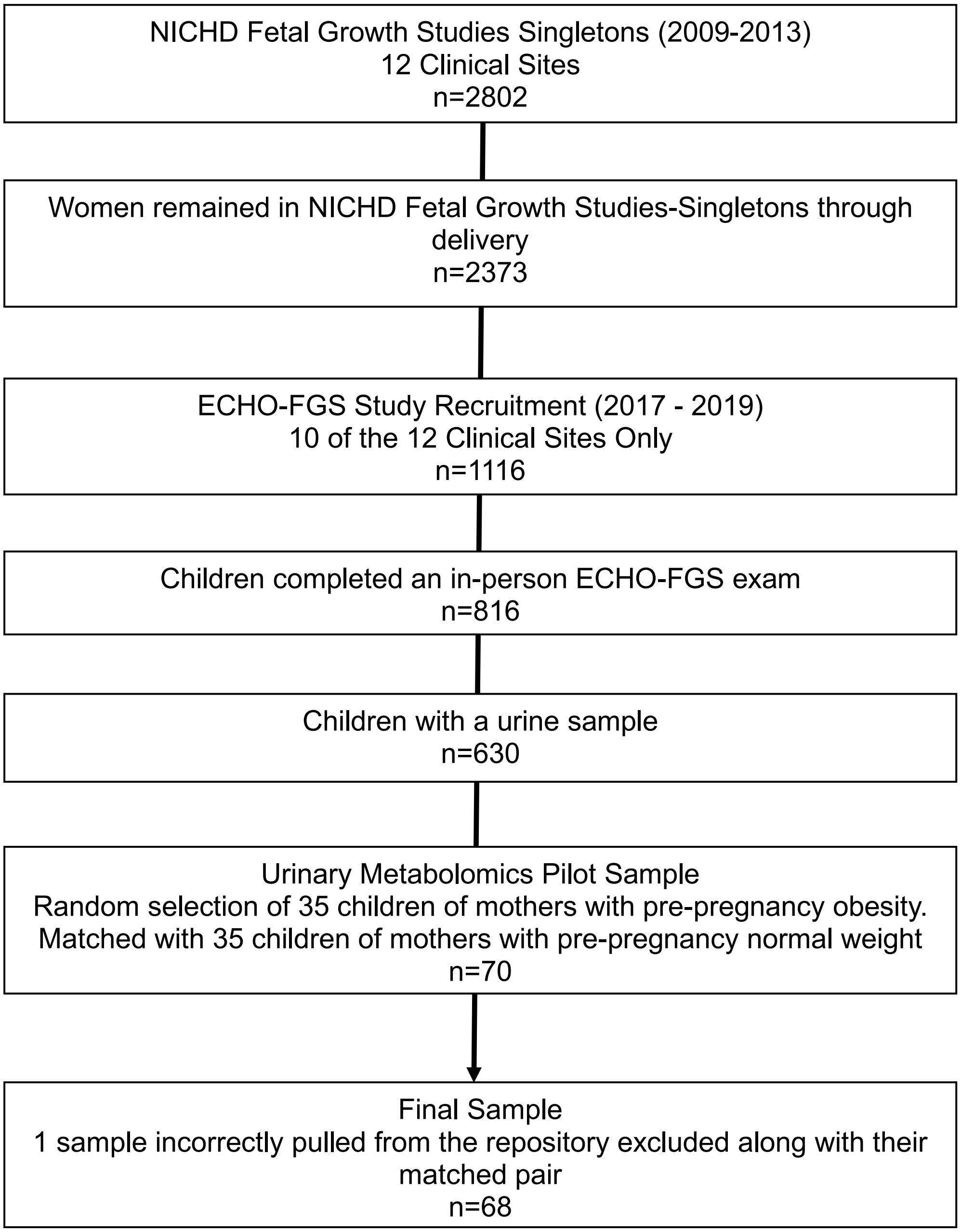

Supplemental Table 2. Weighted correlation network analysis results

Super Pathway	Sub Pathway	Chemical Name	Network Cluster
Lipid	Fatty Acid Metabolism (Acyl Carnitine, Hydroxy)	3-hydroxyhexanoylcarnitine (2)	Blue: metabolite hub based on maternal and offspring BMI categorization mom & child
Lipid	Fatty Acid Metabolism (Acyl Carnitine, Hydroxy)	3-hydroxydecanoylcarnitine	Blue: metabolite hub based on maternal and offspring BMI categorization mom & child
Lipid	Fatty Acid Metabolism (Acyl Carnitine, Hydroxy)	3-hydroxyoctanoylcarnitine (2)	Blue: metabolite hub based on maternal and offspring BMI categorization mom & child
Lipid	Fatty Acid Metabolism (Acyl Carnitine, Medium Chain)	octanoylcarnitine (C8)	Blue: metabolite hub based on maternal and offspring BMI categorization mom & child
Lipid	Fatty Acid Metabolism (Acyl Carnitine, Medium Chain)	2-methylhexanoylcarnitine	Blue: metabolite hub based on maternal and offspring BMI categorization mom & child
Lipid	Fatty Acid Metabolism (Acyl Carnitine, Medium Chain)	cis-3,4-methyleneheptanoylcarnitine	Blue: metabolite hub based on maternal and offspring BMI categorization mom & child
Lipid	Fatty Acid Metabolism (Acyl Carnitine, Short Chain)	acetylcarnitine (C2)	Blue: metabolite hub based on maternal and offspring BMI categorization mom & child
Lipid	Fatty Acid Metabolism (also BCAA Metabolism)	propionylcarnitine (C3)	Blue: metabolite hub based on maternal and offspring BMI categorization mom & child
Amino Acid	Leucine, Isoleucine and Valine Metabolism	2-methylbutyrylcarnitine (C5)	Blue: metabolite hub based on maternal and offspring BMI categorization mom & child
Partially Characterized Molecules	Partially Characterized Molecules	carnitine of C10H14O2 (5)*	Blue: metabolite hub based on maternal and offspring BMI categorization mom & child
Amino Acid	Polyamine Metabolism	N-acetylputrescine	Turquoise: metabolite hub based on maternal and offspring BMI categorization mom & child
Amino Acid	Polyamine Metabolism	(N(1) + N(8))-acetylspermidine	Turquoise: metabolite hub based on maternal and offspring BMI categorization mom & child
Amino Acid	Tryptophan Metabolism	C-glycosyltryptophan	Turquoise: metabolite hub based on maternal and offspring BMI categorization mom & child
Nucleotide	Pyrimidine Metabolism, Uracil containing	pseudouridine	Turquoise: metabolite hub based on maternal and offspring BMI categorization mom & child

Super Pathway	Sub Pathway	Chemical Name	Network Cluster
Nucleotide	Pyrimidine Metabolism, Uracil containing	N3-methyluridine	Turquoise: metabolite hub based on maternal and offspring BMI categorization mom & child
Peptide	Modified Peptides	N,N-dimethyl-pro-pro	Turquoise: metabolite hub based on maternal and offspring BMI categorization mom & child
Amino Acid	Creatine Metabolism	creatinine	Turquoise: metabolite hub based on maternal exposure categorization
Amino Acid	Lysine Metabolism	hydroxy-N6,N6,N6-trimethyllysine	Turquoise: metabolite hub based on maternal exposure categorization
Amino Acid	Methionine, Cysteine, SAM and Taurine Metabolism	N-acetyltaurine	Turquoise: metabolite hub based on maternal exposure categorization
Amino Acid	Urea cycle; Arginine and Proline Metabolism	asymetric dimethylarginine (ADMA)	Turquoise: metabolite hub based on maternal exposure categorization
Carbohydrate	Aminosugar Metabolism	N-acetylglucosaminylasparagine	Turquoise: metabolite hub based on offspring categorization
Nucleotide	Pyrimidine Metabolism, Orotate containing	orotidine	Turquoise: metabolite hub based on offspring categorization
Nucleotide	Pyrimidine Metabolism, Uracil containing	3-(3-amino-3-carboxypropyl)uridine	Turquoise: metabolite hub based on offspring categorization
Amino Acid	Alanine and Aspartate Metabolism	hydroxyasparagine	Turquoise: metabolite hub based on offspring categorization

Supplemental Methods

Metabolomic Processing: The present urine dataset comprises a total of 1,510 biochemicals, 1,028 compounds of known identity (named biochemicals) and 482 compounds of unknown structural identity (unnamed biochemicals). Following batch normalization, imputation of missing values, if any, with the minimum observed value for each compound, and log transformation, a matched pairs *t*-test was used to identify biochemicals that differed significantly between experimental groups.

Sample Preparation: Samples were prepared using the automated MicroLab STAR® system from Hamilton Company. Several recovery standards were added prior to the first step in the extraction process for QC purposes. To remove protein, dissociate small molecules bound to protein or trapped in the precipitated protein matrix, and to recover chemically diverse metabolites, proteins were precipitated with methanol under vigorous shaking for 2 min (Glen Mills GenoGrinder 2000) followed by centrifugation. The resulting extract was divided into multiple fractions: two for analysis by two separate reverse phase (RP)/UPLC-MS/MS methods with positive ion mode electrospray ionization (ESI), one for analysis by RP/UPLC-MS/MS with negative ion mode ESI, one for analysis by HILIC/UPLC-MS/MS with negative ion mode ESI, while the remaining fractions were reserved for backup. Samples were placed briefly on a TurboVap® (Zymark) to remove the organic solvent. The sample extracts were stored overnight under nitrogen before preparation for analysis.

QA/QC: Several types of controls were analyzed in concert with the experimental samples: a pooled matrix sample generated by taking a small volume of each experimental sample (or alternatively, use of a pool of well-characterized human plasma) served as a technical replicate throughout the data set; extracted water samples served as process blanks; and a cocktail of QC standards that were carefully chosen not to interfere with the measurement of endogenous compounds were spiked into every analyzed sample, allowed instrument performance monitoring and aided chromatographic alignment.

Instrument variability was determined by calculating the median relative standard deviation (RSD) for the standards that were added to each sample prior to injection into the mass spectrometers. Overall process variability was determined by calculating the median RSD for all endogenous metabolites (*i.e.,* non-instrument standards) present in 100% of the pooled matrix samples. Experimental samples were randomized across the platform run with QC samples spaced evenly among the injections.

Ultrahigh Performance Liquid Chromatography-Tandem Mass Spectroscopy (UPLC-MS/MS): All methods utilized a Waters ACQUITY ultra-performance liquid chromatography (UPLC) and a Thermo Scientific Q-Exactive high resolution/accurate mass spectrometer interfaced with a heated electrospray ionization (HESI-II) source and Orbitrap mass analyzer operated at 35,000 mass resolution (PMID: 32445384). The dried sample extract were then reconstituted in solvents compatible to each of the four methods. Each reconstitution solvent contained a series of standards at fixed concentrations to ensure injection and chromatographic consistency. One aliquot was analyzed using acidic positive ion conditions, chromatographically optimized for more hydrophilic compounds (PosEarly). In this method, the extract was gradient eluted from a C18 column (Waters UPLC BEH C18-2.1x100 mm, 1.7 µm) using water and methanol, containing 0.05% perfluoropentanoic acid (PFPA) and 0.1% formic

acid (FA). Another aliquot was also analyzed using acidic positive ion conditions, however it was chromatographically optimized for more hydrophobic compounds (PosLate). In this method, the extract was gradient eluted from the same aforementioned C18 column using methanol, acetonitrile, water, 0.05% PFPA and 0.01% FA and was operated at an overall higher organic content. Another aliquot was analyzed using basic negative ion optimized conditions using a separate dedicated C18 column (Neg). The basic extracts were gradient eluted from the column using methanol and water, however with 6.5mM Ammonium Bicarbonate at pH 8. The fourth aliquot was analyzed via negative ionization following elution from a HILIC column (Waters UPLC BEH Amide 2.1x150 mm, 1.7 µm) using a gradient consisting of water and acetonitrile with 10mM Ammonium Formate, pH 10.8 (HILIC). The MS analysis alternated between MS and data-dependent MS^n scans using dynamic exclusion. The scan range varied slightly between methods but covered 70-1000 m/z. Raw data files are archived and extracted as described below.

Bioinformatics: The informatics system consisted of four major components, the Laboratory Information Management System (LIMS), the data extraction and peak-identification software, data processing tools for QC and compound identification, and a collection of information interpretation and visualization tools for use by data analysts. The hardware and software foundations for these informatics components were the LAN backbone, and a database server running Oracle 10.2.0.1 Enterprise Edition.

LIMS: The purpose of the Metabolon LIMS system was to enable fully auditable laboratory automation through a secure, easy to use, and highly specialized system. The scope of the Metabolon LIMS system encompasses sample accessioning, sample preparation and instrumental analysis and reporting and advanced data analysis. All of the subsequent software systems are grounded in the LIMS data structures. It has been modified to leverage and interface with the in-house information extraction and data visualization systems, as well as third party instrumentation and data analysis software.

Data Extraction and Compound Identification: Raw data was extracted, peak-identified and QC processed using a combination of Metabolon developed software services (applications). Each of these services perform a specific task independently, and they communicate/coordinate with each other using industry-standard protocols. Compounds were identified by comparison to library entries of purified standards or recurrent unknown entities. Metabolon maintains a library based on authenticated standards that contains the retention time/index (RI), mass to charge ratio (*m/z)*, and fragmentation data on all molecules present in the library. Furthermore, biochemical identifications are based on three criteria: retention index within a narrow RI window of the proposed identification, accurate mass match to the library +/- 10 ppm, and the MS/MS forward and reverse scores between the experimental data and authentic standards. The MS/MS scores are based on a comparison of the ions present in the experimental spectrum to the ions present in the library spectrum. While there may be similarities between molecules based on one of these factors, the use of all three data points is utilized to distinguish and differentiate biochemicals. More than 5,400 commercially available purified or in-house synthesized standard compounds have been acquired and analyzed on all platforms for determination of their analytical characteristics. An additional 7000 mass spectral entries have been created for structurally unnamed biochemicals, which have been identified by virtue of their recurrent nature (both chromatographic and mass spectral). These compounds have the

potential to be identified by future acquisition of a matching purified standard or by classical structural analysis. Metabolon continuously adds biologically-relevant compounds to its chemical library to further enhance its level of Tier 1 metabolite identifications.

Compound Quality Control: A variety of curation procedures were carried out to ensure that a high-quality data set was made available for statistical analysis and data interpretation. The QC and curation processes were designed to ensure accurate and consistent identification of true chemical entities, and to remove or correct those representing system artifacts, mis-assignments, mis-integration and background noise. Metabolon data analysts use proprietary visualization and interpretation software to confirm the consistency of peak identification and integration among the various samples.

Metabolite Quantification and Data Normalization: Peaks were quantified using area-under-the-curve. For studies spanning multiple days, a data normalization step was performed to correct variation resulting from instrument inter-day tuning differences. Essentially, each compound was corrected in run-day blocks by registering the medians to equal one (1.00) and normalizing each data point proportionately. For studies that did not require more than one day of analysis, no normalization is necessary, other than for purposes of data visualization. In certain instances, biochemical data may have been normalized to an additional factor (*e.g.,* cell counts, total protein as determined by Bradford assay, osmolality, etc.) to account for differences in metabolite levels due to differences in the amount of material present in each sample.
